# Genome-Wide Identification of the *DOF* Gene Family in Kiwifruit (*Actinidia chinensis*) and Functional Validation of *AcDOF22* in Response to Drought Stress

**DOI:** 10.3390/ijms25169103

**Published:** 2024-08-22

**Authors:** Chao Zhao, Hao Bai, Chaoshuo Li, Zhaojin Pang, Lifeng Xuan, Dezhi Lv, Shuaike Niu

**Affiliations:** Biotechnology Laboratory, Shijiazhuang Institute of Pomology, Hebei Academy of Agriculture and Forestry Sciences, Shijiazhuang 05000, China; zhaochao123@nwafu.edu.cn (C.Z.); baihao1997@nwafu.edu.cn (H.B.); chaoshuo1996@foxmail.com (C.L.); hbhsjin@126.com (Z.P.); znlxlf@163.com (L.X.); jimmy844496@163.com (D.L.)

**Keywords:** *Actinidia chinensis*, DOF transcription factors, drought, transcriptional activation

## Abstract

DNA-binding one zinc finger (DOF) transcription factors are crucial plant-specific regulators involved in growth, development, signal transduction, and abiotic stress response generation. However, the genome-wide identification and characterization of *AcDOF* genes and their regulatory elements in kiwifruit (*Actinidia chinensis*) has not been thoroughly investigated. In this study, we screened the kiwifruit genome database and identified 42 *AcDOF* genes (*AcDOF1* to *AcDOF42*). Phylogenetic analysis facilitated the categorization of these genes into five subfamilies (*DOF*-a, *DOF*-b, *DOF*-c, *DOF*-d, and *DOF*-e). We further analyzed the motifs, conserved domains, gene structures, and collinearity of the *AcDOF*gene family. Gene ontology (GO) enrichment analysis indicated significant enrichment in the “flower development” term and the “response to abiotic stress” category. Promoter prediction analysis revealed numerous cis-regulatory elements related to responses to light, hormones, and low-temperature and drought stress in *AcDOF* promoters. RNA-seq expression profiles demonstrated the tissue-specific expression of *AcDOF* genes. Quantitative real-time PCR results showed that six selected genes (*AcDOF04*, *AcDOF09*, *AcDOF11*, *AcDOF13*, *AcDOF21*, and *AcDOF22*) were differentially induced by abscisic acid (ABA), methyl jasmonate (MeJA), and cold, salt, and drought stresses, with *AcDOF22* specifically expressed at high levels in drought-tolerant cultivars. Further experiments indicated that transient *AcDOF22* overexpression in kiwifruit leaf disks reduced water loss and chlorophyll degradation. Additionally, *AcDOF22* was localized to the nucleus and exhibited transcriptional activation, enhancing drought resistance by activating the downstream drought marker gene *AcDREB2A*. These findings lay the foundation for elucidating the molecular mechanisms of drought resistance in kiwifruit and offer new insights into drought-resistant breeding.

## 1. Introduction

Kiwifruit, belonging to the *Actinidiaceae* family, is a perennial vine plant with dioecious characteristics. Kiwifruit is rich in nutrients such as Vitamin C and E, potassium, and fiber, and is highly valued for its rich nutritional content and health benefits. It is known to enhance immunity, promote digestion, and improve skin health [1,2]. Hence, it is widely cultivated as an important fruit tree worldwide [3]. Multiple genomic versions of kiwifruit have been reported to date [4]. Researchers have utilized genomic information to perform detailed functional studies on kiwifruit, including an exploration of molecular mechanisms related to growth, development, and stress resistance, thereby providing essential theoretical foundations for crop improvement and protection [5,6,7].

Transcription factors, a class of proteins that bind to cis-regulatory elements of stress-related genes, regulate gene expression and participate in the plant response to both biotic and abiotic stresses, including drought, salinity, and pathogenic infections [8]. DOF transcription factors, belonging to a subfamily of the zinc finger protein family, usually consist of 200 to 400 amino acids. The highly conserved N-terminal DOF domain, comprising 50 to 52 amino acids, contains a single zinc finger (C-X2-C-X21-32-C-X2-C) structure with a Cys residue and can recognize the typical sequence 5′-AAAG-3′, but this is not exclusive. Additionally, DOF may vary the recognized cis-acting elements depending on the different target genes being regulated [9]. Many DOF proteins have been identified in various plants, including rice [10], eggplant [11], pigeonpea [12], grape [13], banana [14], apple [15], tea [16], durian [17], cherry [18], and pear [19].

DOF transcription factors reportedly play crucial roles in plant seed endosperm development, seed storage, protein synthesis, seed germination, plant defense mechanisms, carbon and nitrogen metabolism-related gene regulation, and, specifically, responses to abiotic stress in plants [9,20,21]. For instance, in maize, *ZmDOF22* is highly induced upon exposure to drought stress and ABA treatment. The ZmDOF22 transcriptional activator contributes to drought tolerance and recovery in maize. Furthermore, studies involving CRISPR/Cas9 and overexpression plants have shown that ZmDOF22 enhances drought resistance by promoting stomatal closure, reducing water loss, and participating in the ABA pathway [22]. In potatoes, the cycling DOF factor 1 (StCDF1) interacts with a long non-coding RNA (lncRNA) named StFLORE and regulates water loss by affecting stomatal growth and diurnal opening. Both StFLORE overexpression and StCDF1 suppression reduce water loss, thereby increasing drought tolerance, demonstrating the significance of StCDF1-StFLORE for vegetative reproduction and water homeostasis [23]. *MdDOF54*, identified in apples, was found to positively regulate drought resistance in apple plants. *MdDOF54*-overexpressing plants exhibit higher photosynthetic rates under prolonged drought stress, compared to wild-type plants. Moreover, *MdDOF54* overexpression leads to a higher survival rate under short-term drought stress, compared to non-transgenic plants. DAP-seq (DNA-affinity purification sequencing) and ChIP-seq (chromatin immunoprecipitation sequencing) analyses have confirmed that *MdDOF54* recognizes cis-elements containing the 5′-AAAG-3′ motif [24]. Plant DOF transcription factors are crucial for regulating responses to non-biological stresses. Thus, we hypothesized that the *DOF* family in kiwifruit might also be involved in the response to abiotic stress.

Several gene families, including the mitogen-activated protein kinase family, are reportedly associated with signal transduction in kiwifruit [25]. *GRAS* genes are reportedly involved in the response to salt stress, and leucine-rich repeat receptor-like proteins (LRR-RLPs) mediate both biotic and abiotic stress [26]. Efforts to identify regulatory factors associated with maturation in the kiwifruit cultivar *A. deliciosa* var. ‘Hayward’ has utilized high-throughput sequencing methods such as sRNA, degradome, and transcriptome analysis. Notably, studies have identified two transcription factors (AdNAC6 and AdNAC7) with the NAM/ATAF/CUC domain as potential targets of miR164, indicating their potential involvement in mediating kiwifruit fruit ripening [27]. Further research on the drought resistance mechanisms of kiwifruit has attracted increased attention from researchers [28,29]. Preliminary research on drought resistance in kiwifruit has been conducted in horticulture. Evaluative experiments by Bao et al. that assessed drought tolerance and recovery capabilities in kiwifruit have simplified evaluation systems, facilitating the identification of rootstocks (MX-1 and HW) well suited for studies on the effects of moderate-to-severe drought stress in kiwifruit. Bao et al.’s [28] experiments have streamlined kiwifruit drought tolerance and recovery assessments, identifying suitable rootstocks like MX-1 and HW for drought stress studies. In addition, research has found that exogenous hormones can induce drought resistance in kiwifruit. For example, melatonin can increase the levels of ascorbic acid (AsA), glutathione, and carotenoids, promoting the growth of kiwifruit seedlings under drought stress conditions [29].

Furthermore, many transcription factors, such as DOF, are reportedly involved in functional studies on kiwifruit, demonstrating the important role of DOF in stress responses. For example, the transient overexpression of *AdDOF3* in kiwifruit led to a significant upregulation of *AdBAM3L*, indicating that the transcription factor AdDOF3 regulates the critical gene *AdBAM3L* involved in starch degradation [30]. However, there is currently no report on DOF transcription factor family-associated genes in kiwifruit, particularly regarding their function in drought resistance. This underscores the need for further exploration into the drought resistance mechanisms of kiwifruit.

Recent advancements in plant genomic sequencing have significantly accelerated the identification of *DOF* genes in various plants, enhancing our understanding of their potential functions in plant stress responses. However, comprehensive studies on the genome-wide structure and function of most *DOF* genes, particularly in kiwifruit, are still needed for further clarification. Despite the completion of kiwifruit genome sequencing over a decade ago, the identification and functional analysis of the kiwifruit DOF transcription factor family remain largely unexplored. This study focused on kiwifruit, utilizing the kiwifruit Hongyang V3 genome [31] as the reference sequence. It encompassed sequence evolution analysis, collinearity analysis, cis-acting elements, GO annotation, RNA-seq expression profiling under various abiotic stress conditions, and the exploration of a potential drought-resistant DOF transcription factor, AcDOF22. These findings are expected to provide a significant foundation for molecular breeding efforts in kiwifruit and for understanding the response to abiotic stresses mediated by AcDOF transcription factors.

## 2. Results

### 2.1. Identification, Characterization, and Phylogenetic Analysis of the DOF Genes in Kiwifruit

Using BLASTP and HMMER (Hidden Markov Models of Evolutionary Reconstruction) search methods, we successfully identified 42 *AcDOF* genes in *Actinidia chinensis* var. ‘Hongyang’. The genes were sequentially designated as *AcDOF1* to *AcDOF42* based on their chromosomal order. The subsequent analysis of the 42 AcDOF proteins revealed that all AcDOFs lacked transmembrane structures and signals, as shown in Table S1. The amino acid numbers and AcDOF protein molecular weights varied significantly from 170 amino acids (AcDOF20) to 1818 amino acids (AcDOF28) and from 19.03 kDa (AcDOF20) to 202.30 kDa (AcDOF28), respectively, with isoelectric points (pI) ranging from 5.76 to 8.44. Isoelectric point analysis revealed values ranging from 4.37 (AcDOF37) to 9.73 (AcDOF06). All protein instability indices were above 40 (Table S1), indicating their instability and susceptibility to degradation. The average hydrophobicity index was less than 0 (Table S1), indicating that all AcDOF proteins were hydrophilic.

**Figure 1:**
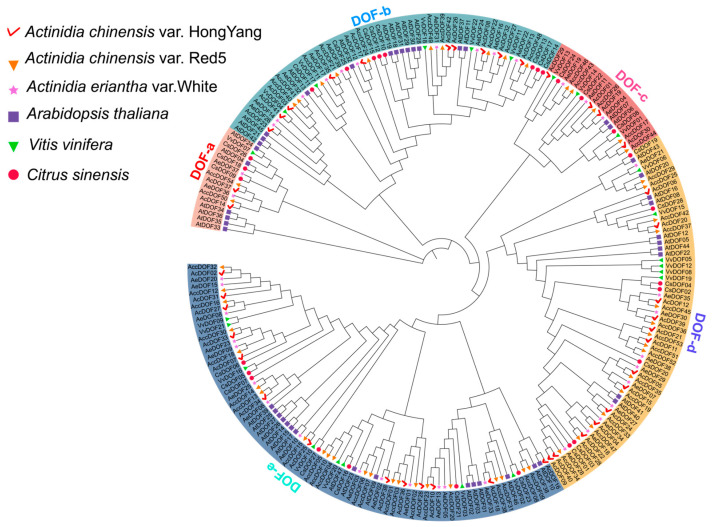
Phylogenetic classification of DOFs in *A. chinensis* var. ‘HongYang’, *A. chinensis* var. ‘Red5’, *A. eriantha* var. ‘White’, *Arabidopsis thaliana*, *Vitis vinifera*, and *Citrus sinensis*. Distinct colors and shapes are employed to distinguish between various species, with color-coded sections on the phylogenetic tree representing five distinct groups. DOF-a, DOF-b, DOF-c, DOF-d, and DOF-e represent different subgroups.

Additionally, using the same methods, we identified DOF family proteins in *A. chinensis* var. ‘Red5’, *A. eriantha* var. ‘White’, *Vitis vinifera*, and *Citrus sinensis* genomes. Among these genomes, *A. chinensis* var. ‘Red5’ had the highest number of identified *DOF* genes at 54, while *A. eriantha* var. ‘White’ had only 39, indicating differences in the number of *DOF* genes among kiwifruit varieties (Table S2). Evolutionary trees based on DOF transcription factors were constructed by identifying *DOF* family genes from the six species mentioned above (Figure 1). The gene tree in Figure 1 was subdivided into five subgroups, each comprising *DOF* genes from six species, with significant differences in the number of *DOF* genes among the subgroups. Additionally, the DOF-a subgroup had the fewest genes, with 16 in kiwifruit. Only one gene (*VvDOF07*) was found in grape in this subgroup, suggesting functional differentiation between kiwifruit and grape. The largest subgroup, DOF-e, contained 76 genes, including 13 *AcDOF* genes from kiwifruit. The DOF-D subgroup had 14 *AcDOF* genes (Figure 1), indicating that the distribution of kiwifruit genes across different subgroups might result from functional diversification. In different clades of the gene evolutionary tree, the three kiwifruit varieties showed closely related evolutionary relationships with no separate branches, indicating the relatively conservative evolution of *DOF* genes among kiwifruit species.

### 2.2. Evolutionary Analysis, Protein Motifs, Conserved Domains, and AcDOF Gene Structure

**Figure 2:**
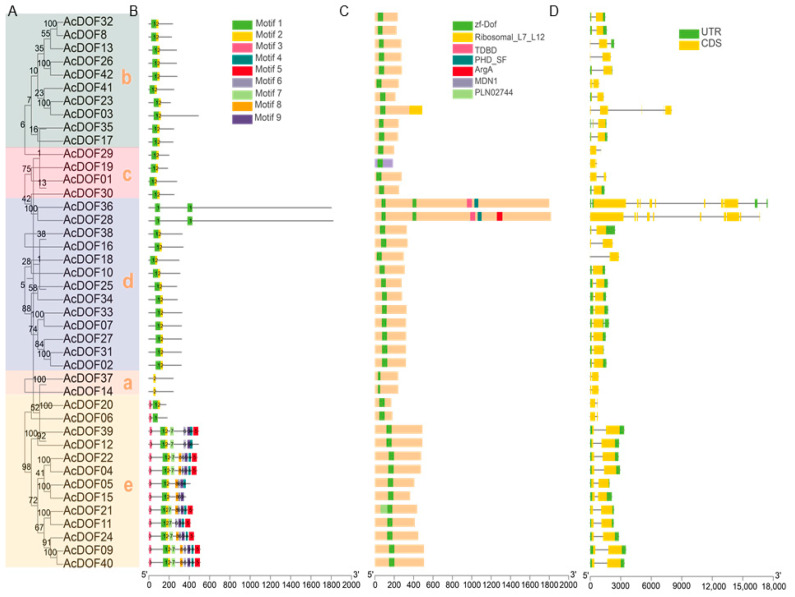
Evolutionary relationships, motif, conserved domains, and gene structure analysis of kiwifruit AcDOFs. (**A**) Kiwifruit AcDOFs evolutionary tree analysis. The lowercase letters (a,b,c,d,e) represent different subgroups. (**B**) Motif analysis. (**C**) Conserved domain analysis. (**D**) Gene structure analysis.

To further investigate the classification, conserved domains, and structure of *AcDOF* genes in kiwifruit, we classified AcDOF family proteins into five subfamilies, each with significant variations based on the evolutionary tree (Figure 2A). Subfamilies b and c both contain motif 1 and motif 2. In subfamily d, except for AcDOF36 and AcDOF28, which contain two copies of motif 1, all proteins contain two motifs (motif 1 and motif 2). In contrast to subfamily d, subfamily a lacks motif 1 in two proteins (AcDOF37 and AcDOF14). Notably, subfamily e exhibits distinct motif characteristics, with all members containing motif 1 and motif 3, except for AcDOF20 and AcDOF06, which contain motif 9 (Figure 2B). This suggests unique motif features among the subfamilies.

Furthermore, conserved domain analysis revealed that all proteins contain the zf-DOF conserved domain (Figure 2C), with AcDOF28 and AcDOF28 also encompassing the TDBD, PHD_SF, and ArgA domains. Finally, gene structure analysis identified *AcDOF36* as the longest gene with a length of nearly 18,000 bp, while *AcDOF01* was identified as the shortest at only 1000 bp. Additionally, variations were observed in the number of introns, with most genes having two to three introns, and a few genes having up to nine introns (AcDOF36) (Figure 2D). In conclusion, structural differences exist among kiwifruit AcDOFs, indicating functional diversity.

### 2.3. Analysis of cis-Regulatory Elements of AcDOFs

**Figure 3:**
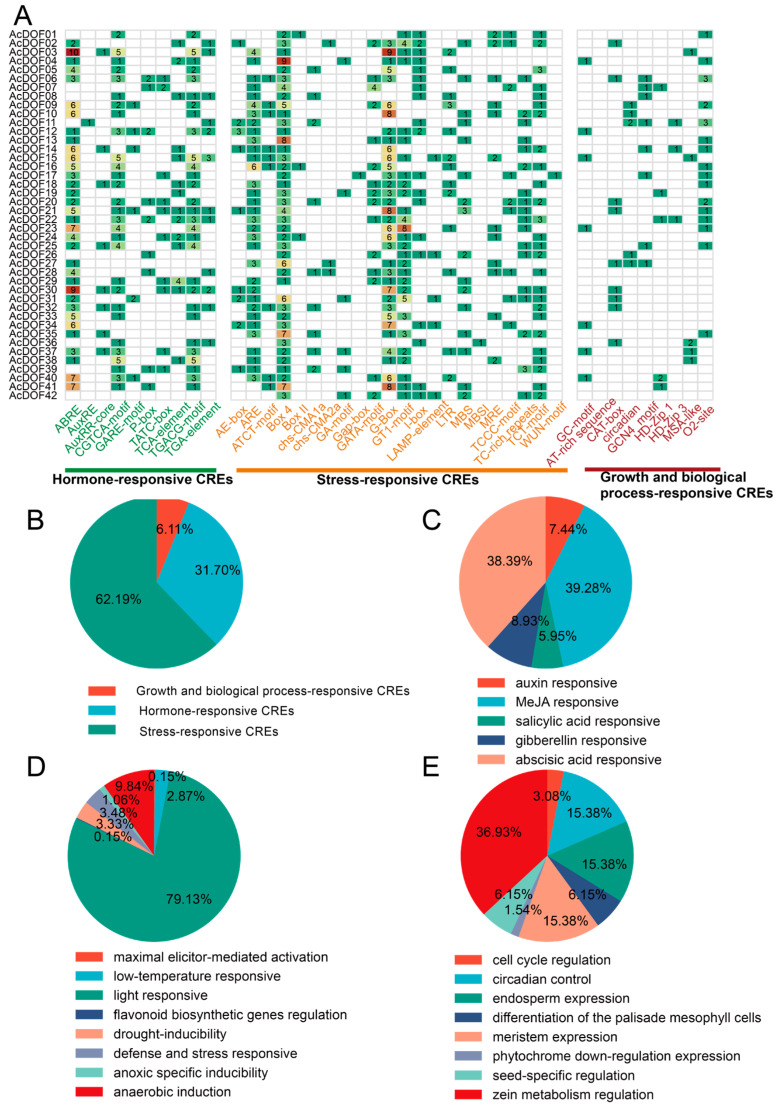
Analysis of cis-regulatory elements in the promoters of 42 AcDOF TFs in kiwifruit. (**A**) Classification of cis-acting elements in kiwifruit *AcDOF* gene family promoters. In the heatmap, green represents smaller values, while red represents larger values. (**B**) The proportions of the three components: hormone, stress-responsive, and growth and biological. (**C**) Hormone-responsive CREs. (**D**) Stress-responsive CREs. (**E**) Growth and biological process-responsive CREs. CREs, Cis-regulatory elements.

To delve deeper into the functional characteristics of the *AcDOF* gene family promoters, this study categorized all cis-elements of the *AcDOF* family into three primary groups based on the functions of each cis-acting element: hormone-responsive cis-regulatory elements (CREs), stress-responsive CREs, and growth and biological process-responsive CREs. Stress-responsive CREs exhibited the highest diversity among the three groups (Figure 3A,B). The promoters of the *AcDOF* gene family encompass various CREs involved in plant stress responses. Notably, the BOX4 element was prevalent in nearly all gene promoters, with the *AcDOF04* promoter showcasing nine BOX4 elements, underscoring the pivotal role of the BOX4 element as a key regulatory component in the ability of kiwifruit to adapt to challenging environments. In hormone-responsive CREs (Figure 3A), the ABRE, AUXRR-Core, and TCA-element CREs were widely dispersed across all genes, indicating the broad involvement of AcDOF-class TFs in hormonal responses. Interestingly, within growth and biological process-responsive CREs (Figure 3B), the proportion of CREs in the promoters of AcDOF TFs was relatively low, comprising only 6.11% of all CREs, while stress-responsive CREs (Figure 3C) and hormone-responsive CREs collectively represented over 30% of all CREs. This suggests that the kiwifruit *AcDOF* gene family might primarily concentrate on responses to environmental stress and hormonal stimuli. Moreover, specific analyses of three main categories were conducted. In hormone-responsive CREs, AcDOF promoters had the most CREs responsive to abscisic acid, followed by MeJA, indicating the involvement of the AcDOF family genes in regulating drought responses in kiwifruit. Light-responsive elements were predominant in stress-responsive CREs (Figure 3D), signifying the vital role of light in kiwifruit growth regulation. In the growth and biological process-responsive CRE category (Figure 3E), CREs related to zein metabolism regulation were most prevalent, underscoring their crucial contribution to growth and development regulation in kiwifruit. The diverse range of CREs in the promoters of the *AcDOF* gene family suggests the multifaceted functionality of kiwifruit *AcDOF* genes.

### 2.4. Collinearity and Synteny Analysis of AcDOFs

**Figure 4:**
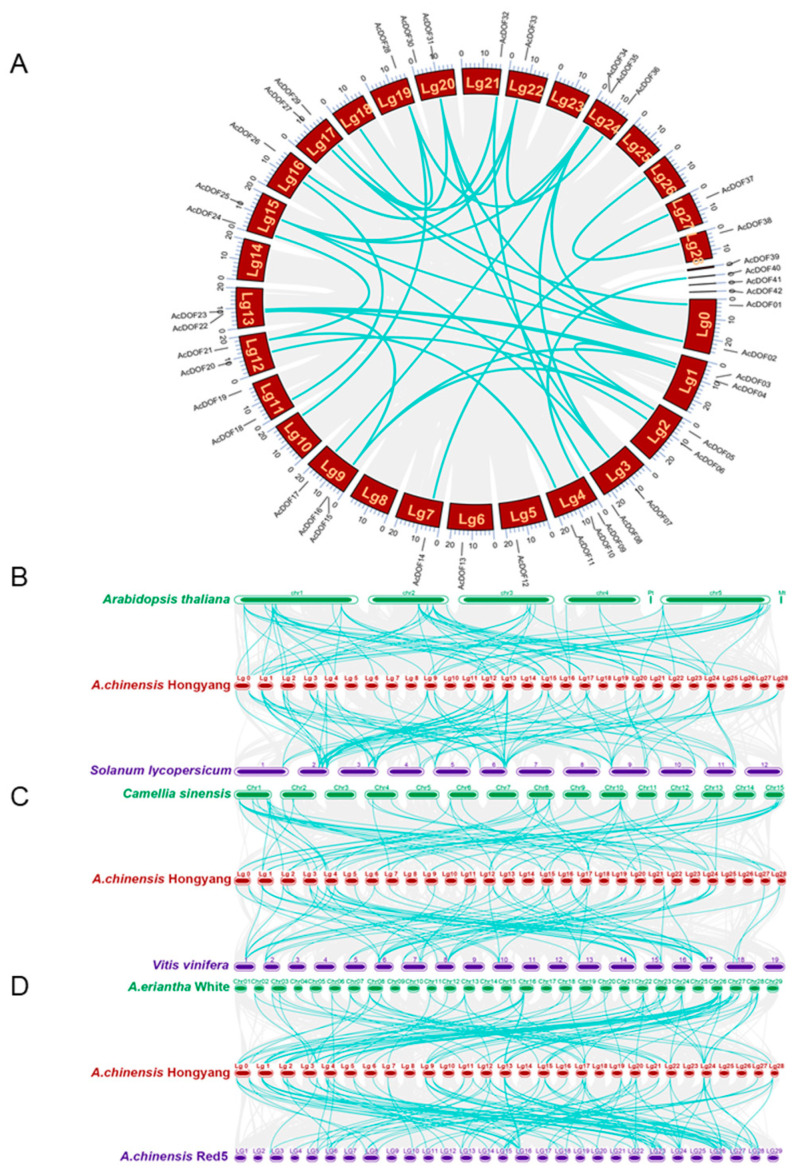
Whole genome duplication and collinearity analysis of *AcDOFs* in kiwifruit. (**A**) Chromosomal location and interchromosomal relationships of *AcDOF* genes in kiwifruit. The identified duplicate events are marked by dark green lines. LG represents the Lachsis group. (**B**) Collinearity analysis between the DOF genes of *Arabidopsis thaliana*, kiwifruit (*A. chinensis* var. Hongyang), and tomato (*Solanum lycopersicum*). (**C**) Collinearity analysis among tea (*Camellia sinensis*), kiwifruit (*A. chinensis* var. Hongyang), and grape (*Vitis vinifera*). (**D**) Collinearity analysis among three different varieties, *A. eriantha* White, *A. chinensis* var. Hongyang, and *A. chinensis* Red5.

This study aimed to investigate the genome-wide duplication events of *AcDOF* genes in kiwifruit and their collinearity relationships with other species. Initially, collinearity analysis was conducted for the *AcDOFs* in the kiwifruit genome. *AcDOFs* were found to be distributed across all chromosomes in kiwifruit except for *Lg5*, *Lg6*, *Lg14*, and *Lg25* (Figure 4A). Additionally, four *DOF* genes were identified on separate contigs rather than specific chromosomes. Furthermore, the distribution of *AcDOFs* varied among different chromosomes, indicating substantial differences in their chromosomal evolution. Collinearity analysis revealed that *AcDOF* genes were distributed across different chromosomes, suggesting that their evolution was dependent on replication events across various chromosomes. Cross-species collinearity analysis showed that the number of collinear gene pairs with *Arabidopsis* (Figure 4B), a model plant, was significantly fewer compared to those in tomato, signifying a closer evolutionary relationship between tomato and kiwifruit. Similarly, in comparisons of tea tree and grape (Figure 4C), minimal differences were noted, with 71 collinear gene pairs in tea tree and 69 in grape. Notably, collinear gene pairs in tea tree predominantly occurred on chromosomes 1 and 8, while those of grape were located on chromosomes 17 and 6. Furthermore, upon comparing *A. eriantha* and *A. chinensis* var. *chinensis* ‘Red5’ (Figure 4D), it was found that the maximum number of collinear gene pairs was 105 in ‘Red5’, whereas the minimum was 86 pairs in *A. eriantha*. These findings suggest potential differentiation among different species.

### 2.5. GO Annotation of the AcDOF Gene Family

**Figure 5:**
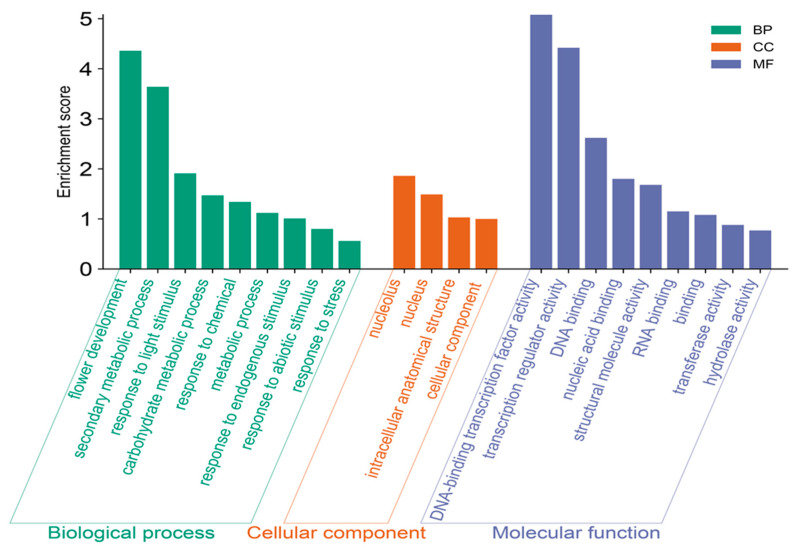
GO functional enrichment of the AcDOFs of kiwifruit.

GO functional annotation can help us understand and interpret the functions and relationships of genes in biological processes [32]. In order to comprehensively understand the functions of kiwifruit AcDOFs, this study annotated all kiwifruit protein sequences using BlastGO software (V.2.2.31), followed by enrichment analysis using TBtools software (V.2.031). The enrichment results of AcDOFs were categorized as biological processes, cellular components, and molecular functions (Figure 5). Within the biological process category, we identified nine highly enriched GO terms, with the highest enrichment score observed for “flower development”, followed by “secondary metabolic process” and “response to light stimulus”, indicating the important regulatory role of DOF TFs in responses of the kiwifruit flower to light stimuli. Additionally, significant enrichments were found in the GO terms “response to endogenous stimulus”, “response to abiotic stimulus”, and “response to stress”, signifying the involvement of AcDOF TFs in regulating the responses of kiwifruit to external abiotic stresses. In the cellular components category, DOF TFs predominantly functioned in the cell nucleus, consistent with the typical characteristics of other TFs. Finally, within the molecular functions category, we observed that “DNA-binding TF activity” exhibited the highest level of enrichment, suggesting that the primary function of kiwifruit DOFs is mediated through DNA-binding interactions. In conclusion, GO annotation analysis revealed the diverse and essential regulatory roles of AcDOFs in kiwifruit, particularly in flower development, responses to light stimuli, and abiotic stress processes through DNA-binding interactions.

### 2.6. Expression Patterns of AcDOFs in Kiwifruit

**Figure 6:**
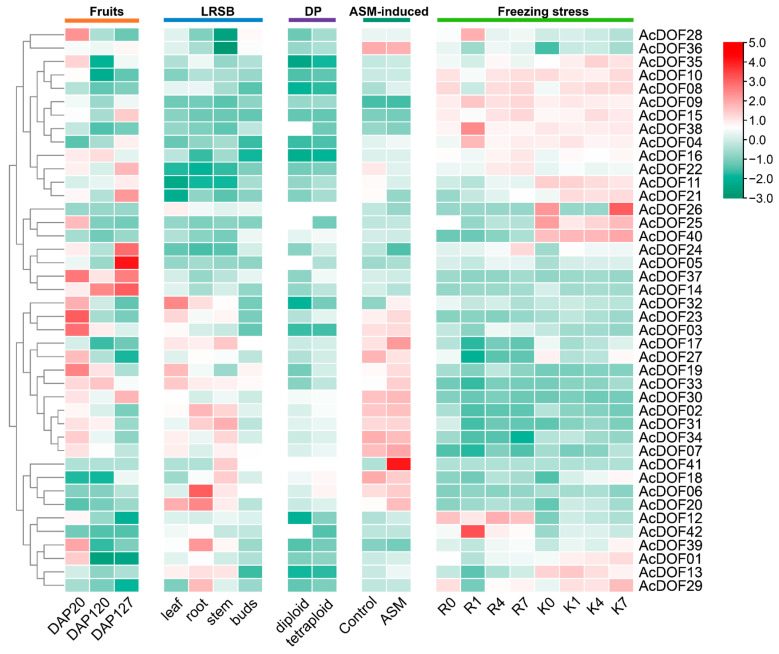
Expression of *AcDOFs* in different tissues, varieties with different ploidy, ASM induction, and freezing stress. DAP, days after pollination. LRSB representing leaf, root, stem, and buds; DP indicating different ploidy; K and R, *A. arguta* genotypes KL (freezing-tolerant) and RB (non-freezing-tolerant); ASM, acibenzolar-S-methyl.

The expression of specific plant genes influences plant traits and their ability to adapt to the environment [33]. Thus, we explored the expression of kiwifruit *AcDOFs* from different perspectives (Figure 6). In fruits, there were significant differences in expression among 42 *DOF* genes, with an overall downregulation observed over time, except for individual genes such as *AcDOF05*, *AcDOF14*, *AcDOF24*, and *AcDOF37*, which showed increased expression at 127 DAP, indicating a more significant role of DOFs during the early fruit development stage. Additionally, in roots, stems, leaves, and buds, approximately half the genes were expressed at high levels across all four tissues, with *AcDOF06* expressed prominently in roots. Expression levels in kiwifruit at different ploidy levels were relatively low but showed differences; for example, *AcDPF38* and *AcDOF42* were expressed at significantly higher levels in diploids compared to tetraploids, suggesting no prominent differences in expression between low- and high-ploidy AcDOFs. Furthermore, the expression of resistance genes in kiwifruit was altered under ASM induction. Genes such as *AcDOF41*, *AcDOF17*, and *AcDOF13* were upregulated under ASM induction, indicating the involvement of some AcDOFs in kiwifruit disease resistance. Finally, gene expression analysis under freezing stress identified multiple highly expressed *AcDOF* genes in cold-tolerant varieties (*A. arguta* genotypes KL), such as *AcDOF25*, *AcDOF26*, *AcDOF29*, and *AcDOF40*, highlighting the crucial regulatory role of AcDOFs in resistance to cold stress. Overall, analyzing the expression characteristics of kiwifruit *AcDOFs* can aid in the discovery of valuable genes.

### 2.7. qRT-PCR Analysis of AcDOFs under Abiotic Stress

**Figure 7:**
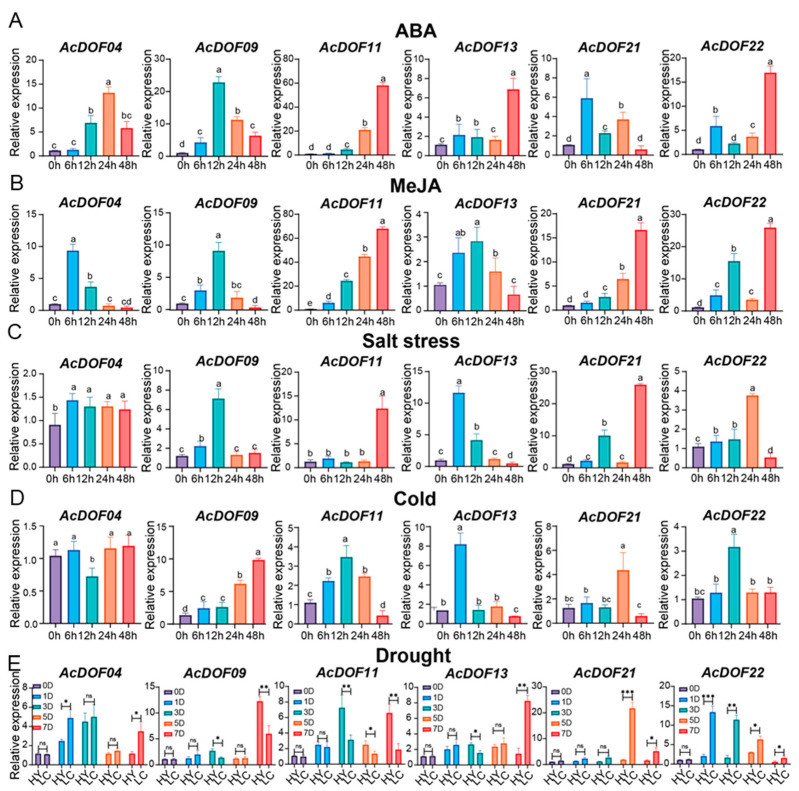
Expression analysis of six *AcDOFs* under abiotic stress. (**A**) The expression of six *AcDOFs* under ABA hormone treatment conditions. (**B**) The expression of six *AcDOFs* under exogenous hormone MeJA treatment conditions. (**C**) The expression of six *AcDOFs* under salt stress. (**D**) The expression of six *AcDOFs* under cold stress conditions. Different letters above the bars indicate a significant difference (*p* < 0.05) determined by the LSD test. (**E**) The expression of six *AcDOFs* under drought stress conditions in drought-resistant (LC—*A. arguta* var. ‘Longcheng NO.2’) and drought-sensitive (HY—*A. chinensis* var. ‘Hongyang’) varieties. Error bars represent the standard deviation of three biological replicates. Student’s *t*-test was used to determine significant differences. * *p* < 0.05; ** *p* < 0.01; *** *p* < 0.001; ns, no significant difference.

Based on the RNA-seq expression profiles and GO enrichment results, we selected 6 out of 16 *AcDOFs* associated with the “response to abiotic stimulus” GO term because of their higher expression levels across all RNA-seq profiles, as candidate genes. Hormone treatments with ABA or MeJA (Figure 7A,B) induced the expression of six *AcDOF* genes. Following ABA treatment, *AcDOF21* showed a rapid response, peaking at 6 h, while *AcDOF22* exhibited a delayed response, still showing an upward trend after 48 h. The MeJA treatment of kiwifruit led to *AcDOF04* showing the quickest response, while *AcDOF21* displayed a delayed response, becoming significantly upregulated after 24 h. This indicates that while these six genes responded to both hormones, their reaction time was varied. The salt treatment of kiwifruit resulted in the differential upregulation of the six genes (Figure 7C), with *AcDOF21* showing the highest fold change (approximately 25-fold at 48 h relative to that at 0 h), while *AcDOF04* exhibited the least significant upregulation (approximately 1-fold).

Additionally, following cold stress treatment, all genes except *AcDOF04* were induced in kiwifruit (Figure 7D). *AcDOF04* was significantly inhibited at 12 h, suggesting its role in balancing gene expression in kiwifruit to prevent excessive imbalance. Furthermore, we observed that *AcDOF13* showed early and positive responses to cold and salt treatments. Finally, we compared the expression of six genes under drought stress in the drought-sensitive HY (*A. chinensis* var. ‘Hongyang’) and drought-resistant LC (*A. arguta* var. ‘Longcheng NO.2’) cultivars and found varying degrees of upregulation in both cultivars (Figure 7E). For instance, *AcDOF04* exhibited significantly higher expression in LC, as compared to HY after 1D, 5D, and 7D of drought treatment (*p* < 0.05). However, *AcDOF11* was expressed at significantly higher levels in HY than in LC after 3D, 5D, and 7D of drought treatment, suggesting its potential negative regulatory role in drought resistance. Notably, except at 0D, *AcDOF22* showed significantly higher expression in LC compared to HY (*p* < 0.05). These results demonstrate the variable induction of the selected six genes under different stress conditions, highlighting their roles in the ABA and JA pathways and suggesting their potential importance as key regulatory factors in abiotic stresses, such as cold, salt, and drought stress.

### 2.8. Subcellular Localization of AcDOF22

**Figure 8:**
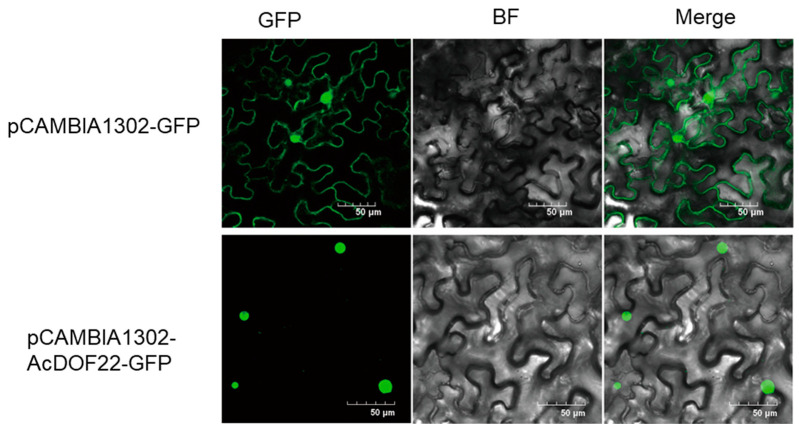
Subcellular localization of AcDOF22. pCAMBIA1302-GFP (empty vector), pCAMBIA1302-AcDOF22-GFP represents the expression vector pCAMBIA1302-GFP fused with the AcDOF22 CDS fragment. Scale bar = 50.0 μm. BF, bright field; GFP, green fluorescent protein.

Based on the results of qRT-PCR analysis, we found that *AcDOF22* responds to hormones, salt, and cold treatments and is expressed at high levels in the drought-resistant cultivar *A. arguta* var. ‘Longcheng NO.2’. Therefore, we conducted further research on *AcDOF22*. To further investigate the intracellular localization of AcDOF22, we separately transformed Agrobacterium GV3101 carrying the pCAMBIA1302-GFP empty vector and the pCAMBIA1302-AcDOF22-GFP recombinant vector, transiently expressed them in the young leaves of *N. benthamiana*, and observed them under a confocal microscope after 48 h. The results showed that control cells emitted green fluorescence signals at both the cell membrane and nucleus (Figure 8), indicating the expression of the pCAMBIA1302-GFP fusion protein in these cellular compartments. In contrast, the AcDOF22-GFP fusion protein predominantly emitted green fluorescent signals in the nucleus, suggesting that AcDOF22 is primarily located in the nucleus, consistent with the characteristics of TFs.

### 2.9. AcDOF22 Positively Regulates Kiwifruit Drought Resistance

**Figure 9:**
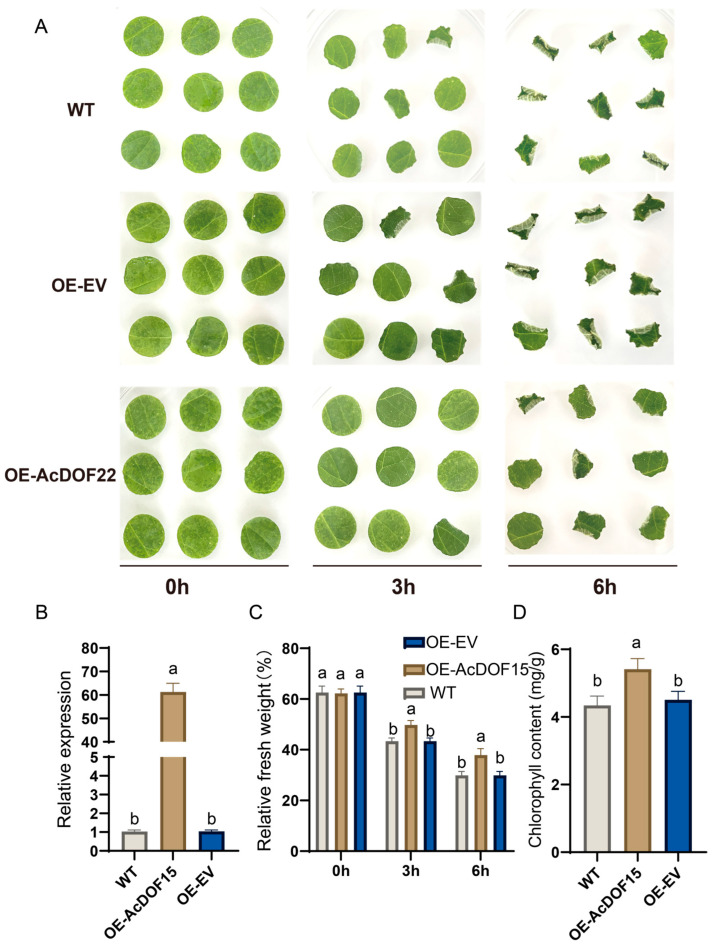
Identification of the drought resistance function of kiwifruit *AcDOF22* gene. (**A**) Transient overexpression in kiwifruit leaf disks. (**B**) qRT-PCR assessment of *AcDOF22* expression levels. (**C**) Relative fresh weight at three time points. (**D**) Measurement of chlorophyll content. OE denotes overexpression, and EV represents empty vector. WT, wild type. Error bars in (**B**–**D**) represent the mean ± standard deviation (SD) of three independent replicates. Different letters above the bars indicate a significant difference (*p* < 0.05) determined by the LSD test.

To further investigate the role of *AcDOF22* in drought resistance, this study employed vacuum infiltration to transiently overexpress *AcDOF22*. The results revealed that there were no discernible differences in leaf disk phenotypes among the control (OE-EV), WT, and experimental (OE-AcDOF22) groups at 0 h. However, after 3 h of drought treatment, mild bending was observed in control leaf disks, while those overexpressing *AcDOF22* exhibited lower levels of bending (Figure 9A,B). Differences became more pronounced after 6 h, with control leaf disks showing significant wilting and shrinkage (Figure 9A). Fresh weight analysis indicated significant differences between the two groups at 3 h and 6 h (*p* < 0.05) (Figure 9C). Furthermore, chlorophyll content measurements demonstrated that under drought conditions, leaf disks overexpressing *AcDOF22* exhibited lower levels of chlorophyll loss (Figure 9D). In conclusion, the experimental findings suggest that *AcDOF22* overexpression in kiwifruit mitigated water loss and chlorophyll degradation, thereby enhancing drought resistance in kiwifruit.

### 2.10. AcDOF22 Transcriptionally Activates AcDERB2A Expression

**Figure 10:**
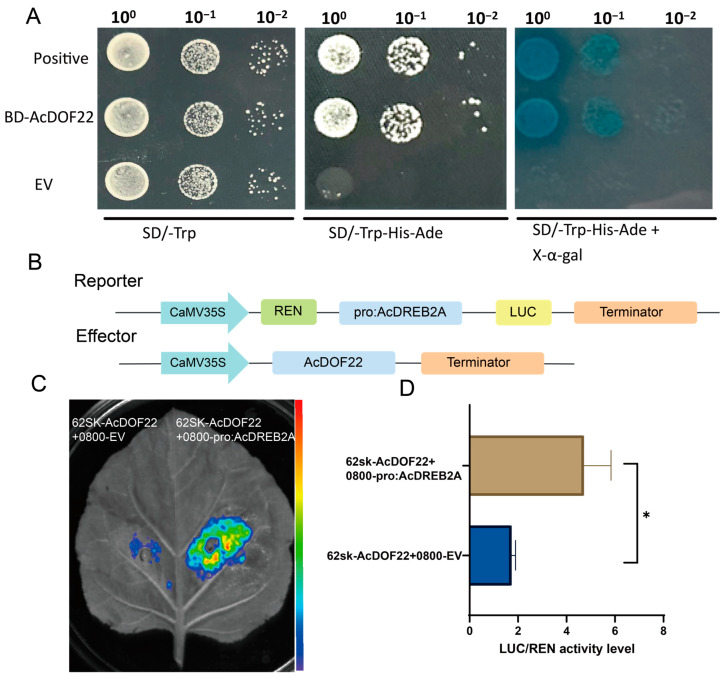
Validating the activation of *AcDREB2A* expression by AcDOF22. (**A**) Transcriptional activation expression verification of AcDOF22. EV, empty vector; BD-AcDOF22 represents the pGBKT7 vector fused with the AcDOF22 CDS segment. Positive refers to pGBKT7-P53. (**B**) Vector construct diagram of effector vector and reporter vector in the dual-luciferase assay. (**C**) Fluorescence images of tobacco leaves in the dual-luciferase assay. The strength of fluorescence in tobacco leaves represents the strength of interaction, with red color indicating a higher interaction intensity. (**D**) Dual-luciferase assay for enzyme activity detection. luc/ren, luciferase enzyme (luc) and renilla enzyme (ren). Error bars indicate the standard deviation of three biological replicates. Student’s *t*-test was used to determine significant differences in relative expression levels at * *p* < 0.05.

The accurately detected BD-AcDOF22 plasmid, BD-P53 (positive control), and BD (empty vector) were separately transformed into yeast Y2H Gold competent cells, spread on SD/-Trp and SD/-Trp/-His/-Ade+X-a-gal agar plates, and incubated at 30 °C in a culture chamber for 2–3 days to observe their growth. The results indicated that BD-AcDOF22, BD-P53, and BD (empty vector) could grow on SD/-Trp agar plates. Moreover, BD-P53 and BD-AcDOF22 not only exhibited growth on SD/-Trp/-His/-Ade plates but also induced blue color in yeast in SD/-Trp/-His/-Ade+X-α-gal agar plates (Figure 10A), indicating the ability of *AcDOF22* to undergo self-activation.

Further analysis revealed that *AcDOF22* is homologous to the *CDF3* gene. *CDF3* can reportedly activate the expression of the downstream drought-resistant marker gene *DREB2A* [34]. To validate whether AcDOF22 in kiwifruit could activate *AcDREB2A* expression, we separately constructed the *AcDOF22* effector vector and the *AcDREB2A* reporter vector (Figure 10B) and conducted dual-luciferase experiments. The results showed that the activity level of the experimental group 62sk-AcDOF22+0800-pro: AcDREB2A LUC/REN was significantly higher than that of the control group 62sk-AcDOF22+0800-pro: AcDREB2A (*p* < 0.05) (Figure 10C,D), indicating that AcDOF22 could activate *AcDREB2A* expression.

## 3. Discussion

DOF transcription factors are widely distributed across the plant kingdom, from lower organisms such as unicellular green algae to higher plants, including angiosperms and gymnosperms [9,20]. Exploring the motifs within AcDOF proteins would enhance our understanding of their unique functions in plant development and stress adaptation. Studies on motif composition have shown that all AcDOF proteins possess motif 1, indicating its conservation and significant role in determining *DOF* gene functions. Similar studies conducted in sweet potato [35] and sunflower [36] found individual motifs in all family members, which aligned with our results, suggesting that this motif may be crucial within the DOF family, playing a pivotal role in *DOF* gene function.

The *DOF* family genes have numerous collinear gene pairs and are relatively conserved in kiwifruit. These results are similar to those reported in sunflower [36]. *AcDOF* genes are unevenly distributed across chromosomes, with abundant replication events occurring among chromosomes, primarily as a single collinearity. This uneven distribution is presumed to result from disparate gene replication on chromosomal segments. Cross-species collinearity analysis shows that DOFs are highly conserved, with numerous collinear gene pairs in both *Arabidopsis* and grape, similar to findings in *Populus simonii* [37] but distinct from sweet potato [35]. This suggests that the number of DOF genes in the genome fluctuates as plants evolve to adapt to environmental changes. In addition, other transcription factors, such as TIFY [38] and ARF [39] have been found to have collinear blocks in *Arabidopsis*, indicating a close genetic relationship. The abundant collinearity of DOF genes in kiwifruit, both within and between species, indicates their evolutionary conservation and suggests functional similarity.

The role of *DOF* genes in responding to abiotic stress has been well documented [20]. This study utilized GO functional annotation and RNA-seq expression profiling to identify six genes (*AcDOF04*, *AcDOF09*, *AcDOF11*, *AcDOF13*, *AcDOF21*, and *AcDOF22*) potentially involved in abiotic stress responses. The JA and ABA signaling pathways play a crucial role in the abiotic stress response [40,41]. The qRT-PCR analysis of six selected *AcDOFs* showed varying responses to both pathways, indicating their involvement in the regulation of JA and ABA signaling. Furthermore, exploring key genes through the combined approach of GO and RNA-seq is very helpful in identifying genes associated with traits.

In addition, *AcDOF04*, *AcDOF09*, and *AcDOF13* were identified as the main early responders in the JA signaling pathway, while *AcDOF11*, *AcDOF21*, and *AcDOF22* were considered late responders. *AcDOF* genes exhibited different expression patterns under various abiotic stresses, with *AcDOF22* being strongly induced under ABA response, and *AcDOF7* significantly induced under low-temperature stress. These findings were similar to studies in rice, where an increased expression of homologous genes (*OsDof19*) [10] was observed in response to cold stress. The results suggest a potential role of these five *AcDOFs* in enhancing cold tolerance in kiwifruit. Furthermore, most genes were activated after stress treatments, probably due to the abundance of CREs in their promoters. Our results underscore the role of *DOF* genes in maintaining the balance in the stress response of *DOF* genes during interactions with the external environment.

DOF transcription factors participate in various plant-specific physiological processes, including light response, seed maturation or germination, tissue differentiation, plant pigment regulation, and metabolism control [9,20,42]. In this study, kiwifruit RNA-seq analysis showed elevated expression levels of *AcDOF* genes in specific kiwifruit tissues, emphasizing their crucial roles in plant growth and development. Furthermore, distinct tissue-specific expression patterns observed in a subset of AcDOF family members suggest specialized functions for these genes. For instance, previous studies have indicated that the DOF protein, DOF AFFECTING GERMINATION (DAG2), acts as a positive regulator in light-regulated seed germination in *Arabidopsis* [43]. In the ‘HuaPi’ fruit of *Fortunella crassifolia*, *FcDof4* and *FcDof16* were significantly correlated with the expression of the flavonoid synthesis gene *FcCGT* during fruit development. Additionally, transient *FcDof4* and *FcDof16* overexpression enhanced the transcription of structural genes in the flavonoid biosynthetic pathway and increased the C-glycosylated flavonoid content [44]. Moreover, in maize, *DOF* genes were involved in starch production. *ZmDOF36* overexpression increased starch levels while reducing soluble sugars, contributing to the development of methods for controlling starch production in the maize endosperm [45]. *AtDOF5.4/OBP4* overexpression in *Arabidopsis* reduced both cell size and number, resulting in dwarf plants. This strongly indicates that *OBP4* acts as a negative regulator of cell cycle progression and cell growth [46].

In GO enrichment analysis, DOF transcription factors were consistently found to be involved in stress responses across several studies. This underscores the diverse functionality of kiwifruit DOF transcription factors under abiotic stress conditions. Previous studies have demonstrated the involvement of *DOF* genes in the drought stress response in potatoes. *StCDF1* was significantly upregulated in the drought-tolerant variety ‘Long10’, while there was no significant change in the drought-sensitive variety ‘DXY’. Similarly, *StCDF2* and *StCDF3* expression levels were decreased in drought-sensitive ‘DXY’ but significantly increased in drought-tolerant ‘Long10’ [47]. Our findings showed that only *AcDOF22* was highly expressed in the drought-tolerant variety (‘LC’), with no upregulation in the drought-sensitive variety (HY). Irregular expression patterns were observed in other *DOFs* in both drought-tolerant and drought-sensitive varieties, suggesting potential drought resistance similar to that observed for *StCDF1* in potatoes. Furthermore, *AcDOF22* was found to significantly enhance kiwifruit leaf drought resistance and decrease water loss. Similarly, the homologous gene *CDF3*, known for its involvement in responses to abiotic stress in plants [34], can enhance drought resistance in *Arabidopsis* by regulating *DREB2A*. *DREB2A*, a key gene in drought conditions, has been widely studied [48]. In plants such as soybean, *GmDof41* overexpression in transgenic hairy roots reduced H_2_O_2_ accumulation and regulated proline homeostasis, thus increasing drought and salt tolerance. *GmDof41* can directly bind to the promoter of *GmDREB2A*, which encodes a DREB2-type protein that affects abiotic stress tolerance in plants [49]. Our study validates the involvement of kiwifruit *AcDOF22* in drought resistance and *AcDREB2A* regulation. Further investigations using techniques such as DAP-Seq or Chip-seq could unveil additional target genes in the *AcDOF22*-regulated drought resistance pathway.

## 4. Materials and Methods

### 4.1. Identification of DOF Gene Family Members and Construction of a Phylogenetic Tree

The genomic and annotation data of *A. chinensis* var. ‘HongYang’, *A. chinensis* var. ‘Red5’, and *A. eriantha* var. ‘White’ were retrieved from the kiwifruit genome website [4] (http://kiwifruitgenome.org/search/genome/5, accessed on 1 June 2023). CDS sequences were extracted and subsequently translated into protein sequences. The complete protein sequences of the *Arabidopsis DOF* gene family were retrieved from the *Arabidopsis* information resource database (http://arabidopsis.org/, accessed on 1 June 2023). Protein sequences of *Vitis vinifera* and *Citrus sinensis* were obtained from EnsemblePlants (http://plants.ensembl.org/index.html, accessed on 22 May 2024). Local blast databases were constructed using BLAST software 2.25 (https://ftp.ncbi.nlm.nih.gov/blast/executables/, accessed on 22 May 2024) for kiwifruit and grape protein sequences, using *Arabidopsis DOF* protein sequences as seed sequences for blastp comparison (threshold < 1 × 10^−5)^. Additionally, the Hidden Markov Model for DOF (PF02701) was downloaded from the Pfam database (http://pfam.xfam.org/, accessed on 22 May 2024). HMMER 3.0 software (threshold < 1 × 10^−5^) was used for the search, along with blastp results. Domain verification was conducted using NCBI Batch Web CD-search (https://www.ncbi.nlm.nih.gov/Structure/bwrpsb/bwrpsb.cgi, accessed on 22 May 2024) and SMART (http://smart.embl-heidelberg.de/, accessed on 5 June 2024). After manual curation, 42 candidate *AcDOF* genes were identified in kiwifruit, following a method similar to that used for grape DOF candidate genes. A multiple sequence alignment of kiwifruit, grape, orange, and *Arabidopsis* DOF protein sequences was performed using ClustalW in MEGAX (https://www.megasoftware.net/, accessed on 5 June 2024), and an evolutionary tree was constructed using the neighbor-joining method with Poisson correction, pairwise deletion, and a bootstrap value of 1000. The evolutionary tree of the *DOF* gene family was visually enhanced using iTOL online software (https://itol.embl.de/, accessed on 5 June 2024). Signal peptide prediction was performed by filtering according to the default settings of the online website.(SignalP 4.0 Server, http://www.cbs.dtu.dk/services/SignalP-4.0/, default parameters, accessed on 5 June 2024), with a D-cutoff value > 0.45 indicating the presence of a signal peptide, otherwise not. The transmembrane structure prediction (TMHMM Server v.2.0, http://www.cbs.dtu.dk/services/TMHMM/, accessed on 5 June 2024) of the *AcDOFs* was conducted using TBtools software (V.2.031) and online resources with default settings [50].

### 4.2. Analysis of the Structural Domains and Conserved Motifs of the Kiwifruit DOF Gene Family

The NCBI CD-Search (https://www.ncbi.nlm.nih.gov/Structure/bwrpsb/bwrpsb.cgi, accessed on 1 June 2024) tool on the National Center for Biotechnology Information (NCBI) website was used to identify conserved domains in the *DOF* gene family. MEME online software [51] (http://meme-suite.org/, accessed on1 June 2024) was employed to predict the conserved motifs of the AcDOF protein family in kiwifruit. TBtools software (V.2.031) was used for visualization [50].

### 4.3. CREs Analysis

In total, 2000 bp upstream DNA sequences of the *AcDOF* gene start codon were extracted from the kiwifruit genome. CREs in the kiwifruit *AcDOF* gene family were predicted using the PlantCare website (http://www.plantcare.co.uk/, accessed on 1 June 2023). Python software (V.3.1.15) was employed for the statistical analysis and visualization of CREs.

### 4.4. Chromosomal Distribution of AcDOF Genes, Duplication, and Synteny Analysis

Chromosome positions of AcDOF family members were obtained based on annotated kiwifruit genome data, and all kiwifruit protein sequences were compared using blastp. MCScanX [52] was subsequently used to analyze tandem gene duplicates in the kiwifruit genome while incorporating genome annotation data. Similarly, the MCScanX software (V.1.1) was used for the collinearity analysis of the *AcDOF* family genes in *Arabidopsis*, kiwifruit, grape, and tea. All collinear regions between species and varieties were determined, and regions containing *AcDOF* genes were selected. Homologous genes in the collinear regions between species and within varieties were considered orthologous genes. The Multiple Synteny Plot module in Tbtools ((V.2.031)) was used to draw collinearity maps between different species and varieties.

### 4.5. GO-Based Annotation of AcDOFs

This study utilized Blast2GO software version 5.0 for functional annotation and GO analysis with default parameters [53]. The simplified operational steps involved extracting the entire CDS sequences of the kiwifruit genome, translating them into protein sequences, and using these sequences as input in Blast2GO software. Subsequently, kiwifruit protein files were aligned against the Swiss-Prot database (https://www.uniprot.org/, accessed on 1 June 2023) using Blast2GO software (threshold < 1× 10^−10^) for comparison using blastp. Then, a functional enrichment analysis of *AcDOF* gene family members was conducted based on the annotation results. The GO annotation outcomes were categorized as molecular functions, cellular components, and biological processes. The results were graphically represented using R software (V.4.0.1, https://www.r-project.org/, accessed on 21 June 2023).

### 4.6. Retrieval of RNA-Seq Expression Profile Data

Transcriptomic data of kiwifruit, encompassing fruit, roots, stems, leaves, ASM induction, and different ploidies, were obtained from the kiwifruit genome website (https://kiwifruitgenome.org/search/genome/5, accessed on 1 July 2024). Short-read archive data for kiwifruit freezing tolerance [54] (Accession number: PRJNA681641; accessed on 1 July 2024) were obtained from the NCBI website, and FPKM values calculated using HISAT2 and DESeq2 were used to estimate gene expression levels. Heat maps were generated using TBtools.

### 4.7. Kiwifruit Plant Treatment, RNA Extraction, and qRT-PCR Analysis

This study utilized two-year-old soft kiwifruit *A. arguta* var. ‘Longcheng No.2’ seedling cuttings, known for their strong stress tolerance when used as experimental materials in Northern China. Following methods established in previous studies [55,56], the seedlings were subjected to salt (NaCl, 200 mM), hormone (0.1 mM ABA and 0.1 mM MeJA), and cold (2 °C) treatments. Two markedly drought-resistant kiwifruit plants, *A. arguta* ‘Longcheng No.2’ (drought-tolerant) and ‘Hongyang’ (drought-sensitive), were used. Three different plant leaves were sampled and combined for each treatment, with 0.15 g of leaf samples being weighed for RNA extraction. RNA extraction was performed according to the instructions provided in the Plant RNA Extraction Kit (ZH120, Huayueyang, Beijing, China), followed by RNA quantification using NanoDrop 2000. RNA sequences with A260/A280 and A260/A230 values ranging from 1.8 to 2.0 ng/μL were deemed suitable for subsequent experiments. RNA integrity was confirmed through electrophoresis on a 0.8% agarose gel. Extracted RNA was reverse-transcribed to cDNA using the HiScript III 1st Strand cDNA Synthesis Kit (R211, Vmayze, Nanjing, China), and PCR amplification was performed using the ChamQ SYBR Color qPCR MasterMix (Q711, Vmayze, Nanjing, China) and an ABI Quantstudio5 Q1 (Thermofisher, Waltham, MA, USA) real-time fluorescence quantifier. The actin gene of kiwifruit was used as an internal reference for relative gene expression level calculations using the ΔΔCT method [57]. The primer pairs used for the qRT-PCR analysis of *AcDOF* are detailed in Table S3.

### 4.8. Subcellular Localization and Yeast Self-Activation

The subcellular localization experiment of kiwifruit *AcDOF22* TF followed a previously established method [58] with slight modifications. First, specific primers for *AcDOF* were designed and the gene was cloned. Subsequently, the linearized pCAMBIA1302 vector was ligated with the AcDOF22 CDS fragment using the ClonExpress II One Step Cloning Kit (C112, Vazyme, Nanjing, China). The correctly assembled vector was transformed into Agrobacterium tumefaciens GV3101 through freeze-thawing, cultured overnight in a shaking culture (28 °C, 200 rpm), adjusted to an OD concentration of 0.6, and injected into tobacco after incubation for 3 h in the dark. The injected tobacco was observed under a laser confocal microscope after 48 h. Additionally, the CDS fragment of *AcDOF22* was seamlessly cloned into the pGBKT7 vector to construct the fusion expression vector pGBKT7-AcDOF22. Following a previously established method [59], the correctly sequenced fusion expression vector was transformed into the Y2HGold yeast strain. Transformed yeast cells were streaked onto yeast-deficient SD/-Trp and SD/-Trp-Ade-His media, incubated in the dark at 30 °C for 48 h, and then photographed for documentation.

### 4.9. Agrobacterium-Mediated Transient Expression in Kiwifruit Leaves

Based on previous research findings [55,60], kiwifruit leaf disk assays were conducted to evaluate the drought resistance function of kiwifruit genes. Initially, the fusion overexpression vector pCAMBIA1302-AcDOF22 carrying the *AcDOF22* CDS fragment was transformed into Agrobacterium tumefaciens GV3101. The primers used for constructing the vector are found in Table S3. Subsequently, a solution for cell infection was prepared, by adjusting the bacterial concentration until OD600 = 0.6. Leaf disks were immersed in the infection solution and vacuum-infiltrated for 10 min, and then dried with absorbent paper. First, the cells were cultured on a 0.7% agar medium for 48 h (25 °C) and leaf disks were placed in a chamber under constant temperature and humidity and light illumination (25 °C). Samples were collected and photographed at 0, 3, and 6 h after incubation.

### 4.10. Dual-Luciferase Assay

The dual-luciferase assay was performed as described previously [61,62]. The *AcDREB2A* promoter (Table S4) and *AcDOF22* CDS fragments were fused with the pGreenII 0800-LUC vector and pGreenII 62-SK linear vector to generate the reporter and effector constructs, respectively. Recombinant plasmids were separately transformed into Agrobacterium rhizogenes GV3101, followed by co-infiltration into tobacco leaves with a 1:1 mixture (OD600 = 0.6, *v*/*v*) of the reporter and effector Agrobacterium strains. The LUC/REN activity was assessed using the Dual-Luciferase Reporter Assay System (Promega, Madison, WI, USA). The primers used for constructing the vector are found in Table S3.

## 5. Conclusions

We conducted a systematic phylogenetic comparative analysis of 42 *DOF* genes in kiwifruit, which revealed the diversity and conservation of these genes within the kiwifruit *DOF* gene family. In addition, we conducted a comprehensive analysis of motifs, conserved domains, and gene structures, and whole-genome duplication analysis. Investigations into CREs indicated the presence of various light-responsive and hormone-responsive regulatory elements in the promoter region of *AcDOFs*. Furthermore, RNA-seq analysis showed that *AcDOF* genes exhibit both tissue-specific and differential expression across ploidy levels in kiwifruit. GO function enrichment analysis demonstrated the association of *AcDOFs* with processes related to flowering, light responses, and abiotic stress responses. Expression pattern analysis via qRT-PCR revealed the differential involvement of six *AcDOFs* in kiwifruit responses to drought, cold, salt, and hormones. Subsequently, we selected the highly expressed drought-resistant gene *AcDOF22* from drought-tolerant varieties and overexpressed it in kiwifruit leaves, demonstrating its positive effect on kiwifruit drought tolerance. Moreover, its localization in the nucleus and the activation of *AcDREB2A* expression were identified as key mechanisms for regulating drought responses. In summary, this study lays the foundation for the involvement of DOF transcription factors in regulating drought resistance in kiwifruit.

## Data Availability

The data and materials that support the findings of this study are available from the corresponding authors upon reasonable request.

## Supplementary Materials

The following supporting information can be downloaded at: https://www.mdpi.com/article/10.3390/ijms25169103/s1.
