# Supplementary material for: Genome-Wide Identification of the DOF Gene Family in Kiwifruit (Actinidia chinensis) and Functional Validation of AcDOF22 in Response to Drought Stress
Source: Int J Mol Sci. 2024 Aug 22;25(16):9103. doi: 10.3390/ijms25169103 (PMC11354610; doi:10.3390/ijms25169103)
Supplement: Supplementary file 1 [file ijms-25-09103-s001.zip › TableS4.pdf]

TableS4.The promoter sequence of AcDREB2A

>AcDREB2A(Actinidia00973) promoter sequence

Note: The sequence marked in blue (5’-AAAG-3’) is a potential binding site for AcDOF22.

5’-ACTATATCTATAAAGTAAAGCTAAAAAAATTTGGCTTTTTTAATAGTAAATTTTAAAAAATTT
AATTTTTAAGCATTACCAAACATAGCCTAAGTTACTCTTTGCTCTACGAATTATGCACTTGC
GTAATTAAGGATGATACACATGAACCAATCAAACTACTCGAGGCTAAATATTCGTCAAATTC
ATTGATAAATAAATTAAAAAAATATATTATTAATAATAAAATTATAGATTCTATTACAAATGAGTA
TACATAGTTTCCCTAGTAAAATCATATTTTTAAGGGGCTCATAAAATACGAGGGTCCATTCA
CACCCCCACACACCACATAGTCCCCACCTGTGGCCCCCGGATATTTTACGTTCCAGCGTG
TGTGTGTGTGATCACACAGGGTTGGGTGGAACCGTGGTGTGTCTAATCCTACAAAAATTT
TAAATTAACTATTTAAATACTTTACCGATTCGAATTGGGGATAAGGTTATAAATGCTTAAAATT
TTTTAATGAAATTGAGTTTCGTTTTATGATCACGCGTTCTCGTTCGAAATTGATGCGTTTAAT
AAATAAATCAAATTCAATATTTTGTTAAAAAGTTATTATGCTTTAAATCAAGTTCGAACAATTT
ACTTATTAACCGTACGGAAAATTAGGTTGAAATATAGGGTATGTTTTTTAAAGAAATAAGGGT
TGTAATATTAATATCCAATATTTACTTGTGGTGGTAATATTAAAAGATGAAAAATATCGAGTGC
TTTCGCGATCCTTCATCCATGAGCAATTATCAGGTCTAGCACGTTCTACAACCTCCCGATG
ACTATCTACACGTGGCACATTTCCAGGCAACCTTCCGTAATTTGACATCAATGGCACAATT
GAAATTTAAATGGAACACGAGGGCAAAGTATAACCCTGGGGGGTAAAACAGGAATACTTC
GTTGCCAAGATAGCAATGTTCCGTTGCATCGAGGTTCGGGAAACTTCGTGCGAAACGGAT
CAGGATCCTGGATATTTTGGTGCAGTTTTACGACGTGGTCTGGTACACTCAGTAACACTTG
CACTTAAATATCAGTAATCCTTATCCGTGGGAAAAGTGTTGGCGCGTTCGGTACAGCGATT
AGTATTCCTCCACGTCATCAATCCGAATAGTAAAAAAATAAAAATATCTACATTTATGTCTTC
GTCATGAAATATCTTCAGGATTCTATAAATTCCCCAGCAGCTGCGCGTGAAAGAGAT-3’
